# Cryo-EM structures of amyloid-β and tau filaments in Down syndrome

**DOI:** 10.1038/s41594-024-01252-3

**Published:** 2024-03-29

**Authors:** Anllely Fernandez, Md Rejaul Hoq, Grace I. Hallinan, Daoyi Li, Sakshibeedu R. Bharath, Frank S. Vago, Xiaoqi Zhang, Kadir A. Ozcan, Kathy L. Newell, Holly J. Garringer, Wen Jiang, Bernardino Ghetti, Ruben Vidal

**Affiliations:** 1 Department of Pathology and Laboratory Medicine, Indiana University School of Medicine, Indianapolis, IN USA; 2 Department of Biological Sciences, Markey Center for Structural Biology, Purdue University, West Lafayette, IN USA; 3 Stark Neurosciences Research Institute, Indiana University School of Medicine, Indianapolis, IN USA

**Keywords:** Cryoelectron microscopy, Diseases

## Abstract

Adult individuals with Down syndrome (DS) develop Alzheimer disease (AD). Whether there is a difference between AD in DS and AD regarding the structure of amyloid-β (Aβ) and tau filaments is unknown. Here we report the structure of Aβ and tau filaments from two DS brains. We found two Aβ_40_ filaments (types IIIa and IIIb) that differ from those previously reported in sporadic AD and two types of Aβ_42_ filaments (I and II) identical to those found in sporadic and familial AD. Tau filaments (paired helical filaments and straight filaments) were identical to those in AD, supporting the notion of a common mechanism through which amyloids trigger aggregation of tau. This knowledge is important for understanding AD in DS and assessing whether adults with DS could be included in AD clinical trials.

## Abstract (web summary)

Here, using cryo-EM, authors reveal that amyloid-β and tau are identical in Alzheimer disease and Down syndrome. This has implications for assessing whether adults with Down syndrome could be included in Alzheimer disease clinical trials.

## Main

Down syndrome (DS) is the most common and best-known chromosomal disorder in humans and the most frequent cause of intellectual disability of genetic origin, affecting about 6 million people worldwide^1,2^. DS is caused by the presence of an extra full or partial copy of chromosome 21 (*Homo sapiens* autosome 21 or HSA21)^3,4^, and hence is also called trisomy 21. Individuals with DS are diagnosed with Alzheimer disease (AD) by the age of 55–60 years, and sometimes as young as 40 years^5–15^. Triplication of the amyloid-β (Aβ) precursor protein (*AβPP*) gene, located on chromosome 21 (21q21.2-3), results in AβPP overproduction in DS, with levels of *AβPP* messenger RNA that are elevated over 1.5-fold compared to controls^16^. Rare DS cases bearing only partial trisomy, excluding the *AβPP* locus, show little or no AD-type pathology, even at advanced age^5,17,18^. Duplications at the *AβPP* locus resulting in AβPP overproduction in individuals without DS have been reported in families with AD^19^, strongly supporting the role of Aβ in the pathogenesis of AD in DS.

The AD neuropathological phenotype observed in individuals with DS, which includes Aβ deposition (parenchymal and vascular) and neurofibrillary tangles (NFTs), is like that described in patients with AD^10,19,20^; however, variations between AD in DS and AD caused by *AβPP* locus duplications have been reported, suggesting that additional genes/factors may modulate the development of AD in DS^19^. Most neuropathologic studies comparing AD and AD in DS have focused on the characterization of Aβ in amyloid plaques and cerebral amyloid angiopathy (CAA), the spread of Aβ and tau pathology and the presence of copathologies^5,20–23^. Aβ is a 40- or 42/43-amino acid peptide generated by the successive proteolysis of AβPP by the β-site AβPP-cleaving enzyme 1 (BACE1) and the γ-secretase complex^24^. After β-cleavage, the carboxyl terminal fragment of AβPP, known as CTFβ, remains membrane associated and is further cleaved by γ-secretase releasing Aβ species of varying lengths^25^. In the brain parenchyma, Aβ peptides are the main component of senile plaques and diffuse deposits. Senile plaques contain a heterogeneous mixture of peptide species comprising Aβ1–42 (Aβ_42_) and Aβ1–43 (Aβ_43_), amino-terminally modified and truncated Aβ peptides ending at positions 42 and 43, and post-translationally modified Aβ peptides^26^. Diffuse deposits, which are mostly found in the cerebral and cerebellar cortices, neostriatum and hypothalamus, also contain full-length and amino-terminally modified and truncated Aβ peptides ending at Aβ_42_ and Aβ_43_ (ref. ^27^). In the vascular compartment, Aβ deposition is found in the walls of large and small leptomeningeal vessels, as well as intraparenchymal medium-sized and small vessels. Aβ_40_ is the predominant Aβ peptide, with carboxyl terminal-truncated derivatives found in both leptomeningeal and cortical vessels^26^. Filamentous tau inclusions occur in the form of NFTs in cell bodies, neuropil threads in the processes of nerve cells and dystrophic neurites associated with Aβ plaques in the cerebral cortex. Tau filaments are composed of six tau isoforms, having three isoforms with three microtubule-binding repeats (3R tau) and three isoforms with four microtubule-binding repeats (4R tau)^28^.

**Extended Data Fig. 1:**
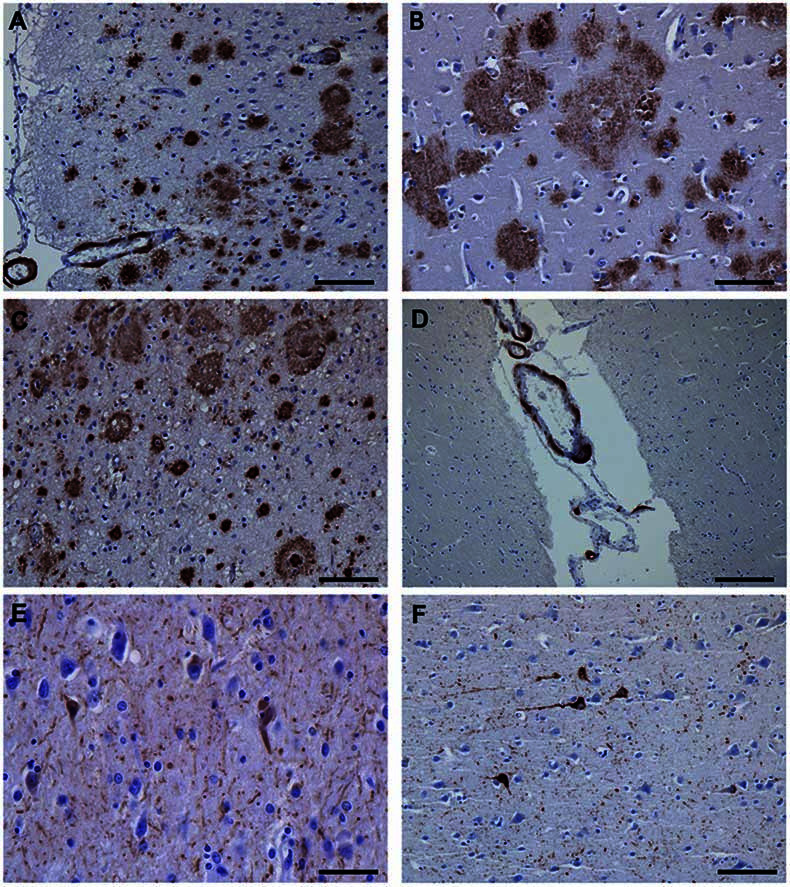
Immunohistochemistry. Immunohistochemical staining of sections of the frontal cortex (DS case #1, panels A, C, E) and temporal cortex (DS case #2, panels B, D, F), contralateral to the ones used for isolating amyloid fibrils, using antibodies specific for Aβ (**a–d**) and tau (E, F). A significant number of neuritic and diffused plaques can be observed using antibody 4G8 (A, B) and the C-terminal specific Aβ antibody against Aβ species ending at position 42 (C). Vascular amyloid deposition is seen using 4G8 (A) and the C-terminal specific Aβ antibody against Aβ species ending at position 40 (D). Presence of tau aggregates can be seen using the anti-tau antibody AT8 (E, F). Scale bars, 100 μm (A-C, F), 200 μm (D), and 50 μm (E).

The recent characterization by cryo-electron microscopy (cryo-EM) of the structure of Aβ and tau filaments in AD and related disorders^29–31^ allows researchers to gain further insights into similarities and differences between AD and AD in DS, by comparing the structure of Aβ and tau filaments between the two. In this Article, we describe the use of cryo-EM to characterize Aβ and tau filaments extracted from the brains of two individuals with DS. Presence of AD pathology in both cases was neuropathologically confirmed (Extended Data Fig. 1). Trisomy 21 was verified by chromosomal microarray analysis on genomic DNA from brain tissue. Our work allows for comparisons between the structures of Aβ and tau filaments in AD versus AD in DS and provides insights in the pathogenic mechanisms that may lead to AD in DS. This information may be crucial for the inclusion of people with DS in prevention trials of AD.

## Results

### Structure of Aβ filaments ending at Ala_42_ (Aβ_42_) in DS

**Fig. 1:**
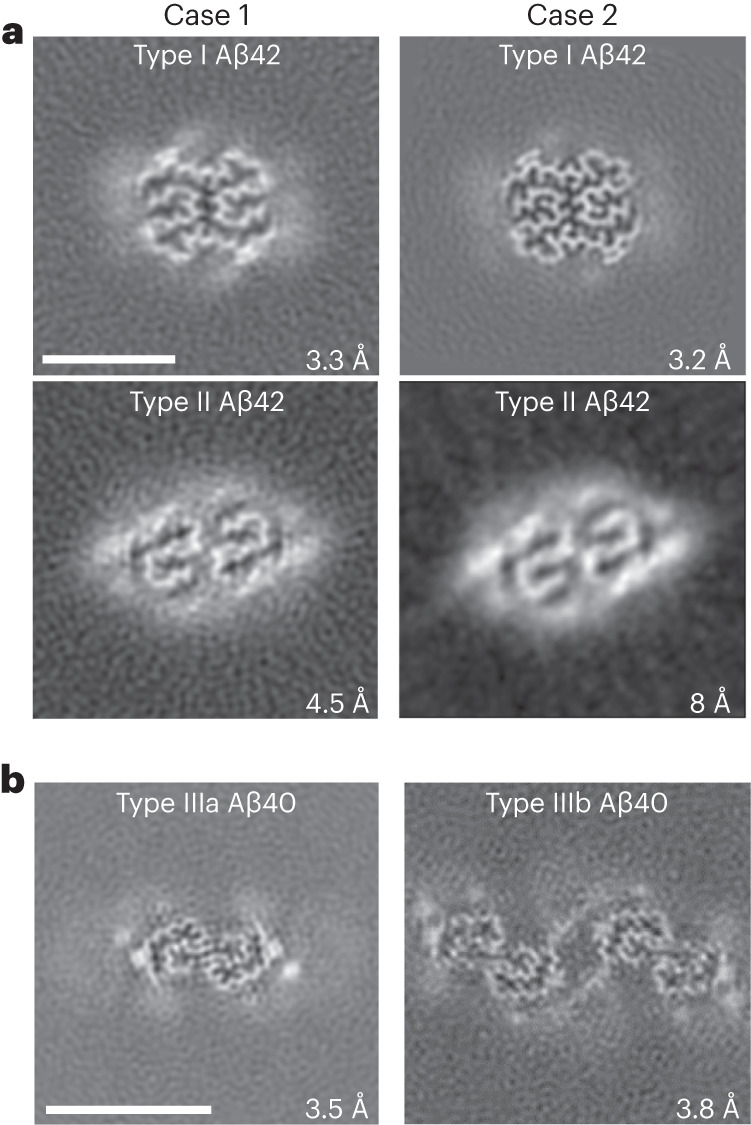
Cryo-EM reconstructions of Aβ filaments. **a**, Cryo-EM maps, depicted as the sum of ~5 Å central *Z*-slices, of Aβ_42_ filaments in DS cases 1 and 2. **b**, Case 2 type IIIa and IIIb Aβ_40_ filaments. The estimated resolution is shown on the bottom right. Scale bar, 5 nm (**a** and **b**). A total of 12,540 and 24,881 movies were collected for cases 1 and 2, respectively.

**Fig. 2:**
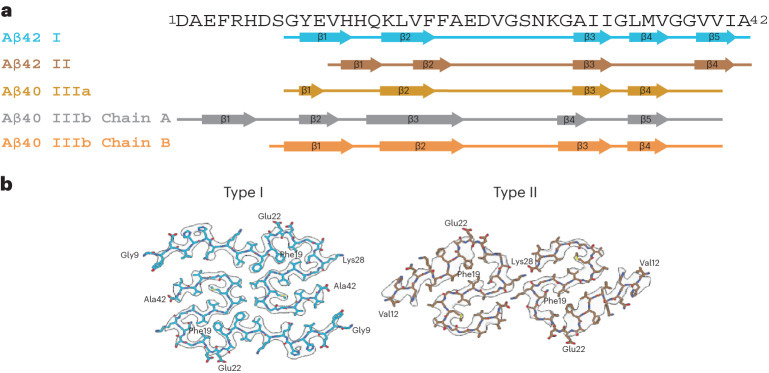
Aβ filaments in DS brain. **a**, Aβ amino acid sequence showing the location of the β-strand regions in Aβ_42_ type I and II protofilaments and in Aβ_40_ type III protofilaments. Type I Aβ_42_ filaments and type II Aβ_42_ filaments comprise Gly_9_ to Ala_42_ and Val_12_ to Ala_42_, respectively. Type IIIa Aβ_40_ filaments are composed of two pairs of identical protofilaments that comprise residues Gly_9_ to Val_40_. Type IIIb Aβ_40_ filaments are organized as a dimer of dimers composed of two pairs of nonidentical protofilaments that comprise residues Ser_8_ to Val_40_ and Asp_1_ to Val_40_. **b**, Cryo-EM map in transparent gray, with an atomic model of a single molecule of type I and type II Aβ_42_ filaments in DS.

**Extended Data Fig. 2:**
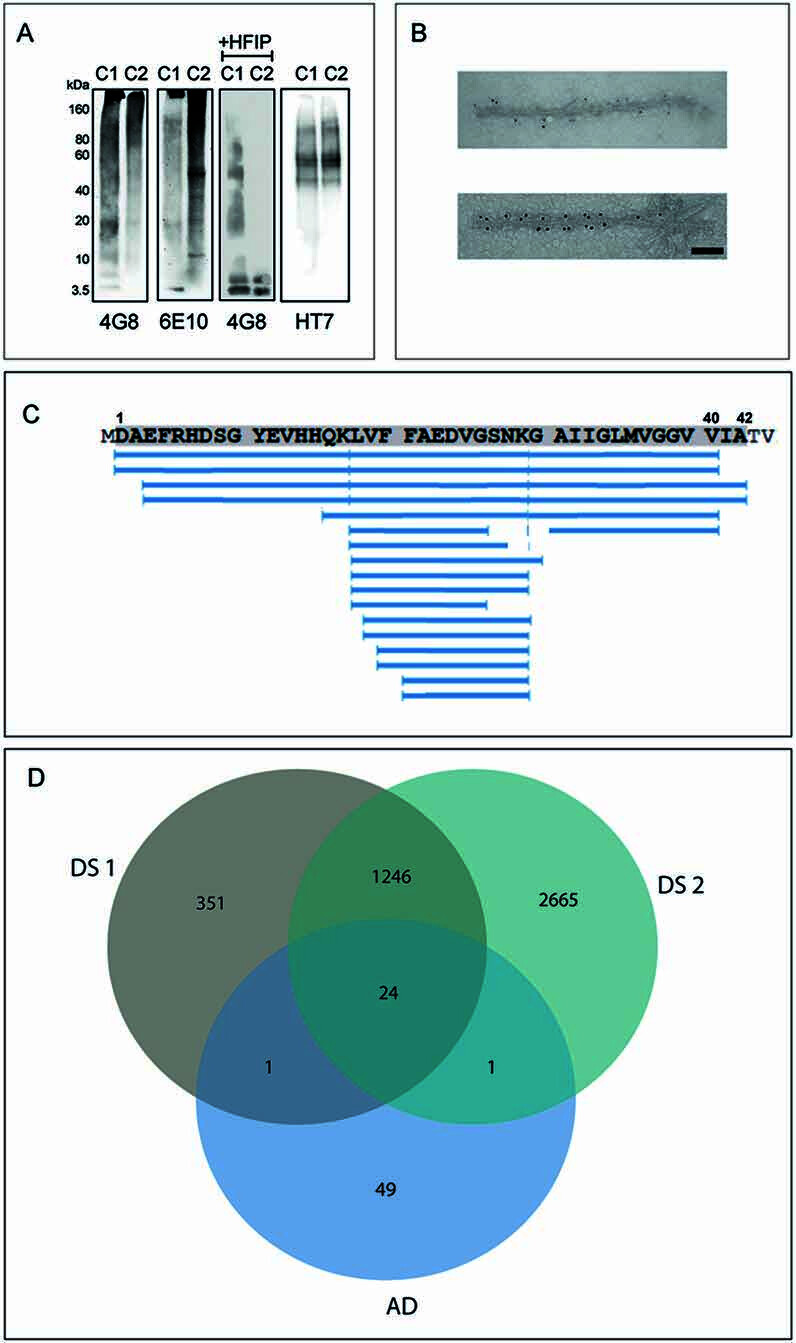
Biochemical analysis of Aβ and tau in DS cases 1 and 2. (**a**) Western blot shows the presence of Aβ peptides and tau isolated from the cerebral cortex from cases #1 and #2. Presence of Aβ was assessed using antibodies 4G8 and 6E10. Samples were treated with HFIP (+HFIP) to disaggregate Aβ fibrils before western blot using 4G8. Presence of tau was assessed using antibody HT7. Molecular masses are in kDa. This experiment was performed three times. (**b**) Representative images of immuno-TEM of sarkosyl insoluble Aβ filaments labeled by 6 nm immunogold particles and tau filaments labelled by 10 nm immunogold particles. Scale bar, 100 μm. This experiment was performed three times. (**c**) Mass Spectrometric analysis showing coverage of the Aβ peptide sequence after trypsin digestion. (**d**) Several proteins were identified in both DS cases (1,268), with 24 unique hits present in DS cases and in AD (32). This experiment was performed one time. Source data

**Extended Data Fig. 3:**
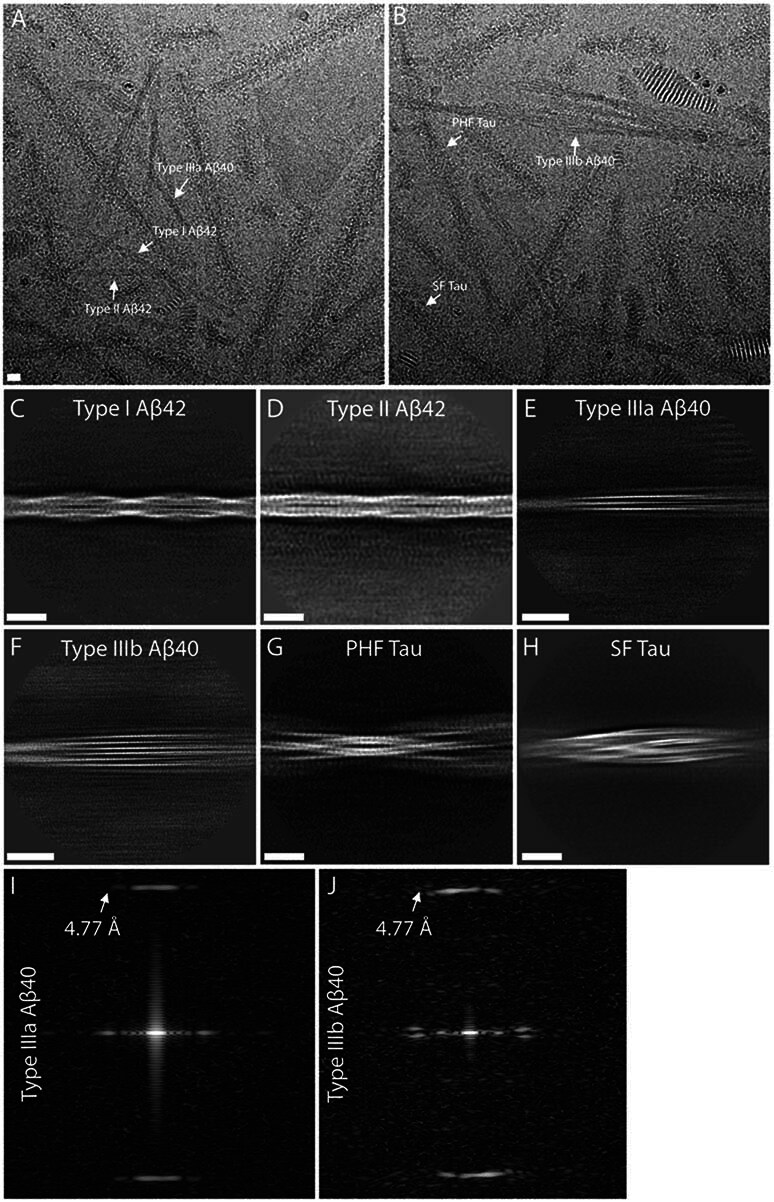
Representative cryo-EM images of filaments from sarkosyl-insoluble fractions from case #2. (**a**, **b**) Blue arrows indicate type I and purple arrows indicate type II Aβ_42_ filaments in two raw micrographs. Green arrows indicate type IIIa and red arrows indicate IIIb Aβ_40_ filaments. White and yellow arrows indicate PHF and SF tau filaments, respectively. (**c–h**) 2D class averages of type I, II, IIIa and IIIb Aβ filaments, and PHF and SF tau filaments. (**i–j**) Power spectra of IIIa and IIIb Aβ_40_ filaments 2D class averages. The scale bars represent 10 nm. A total of 24,881 movies were collected for case #2.

**Extended Data Fig. 4:**
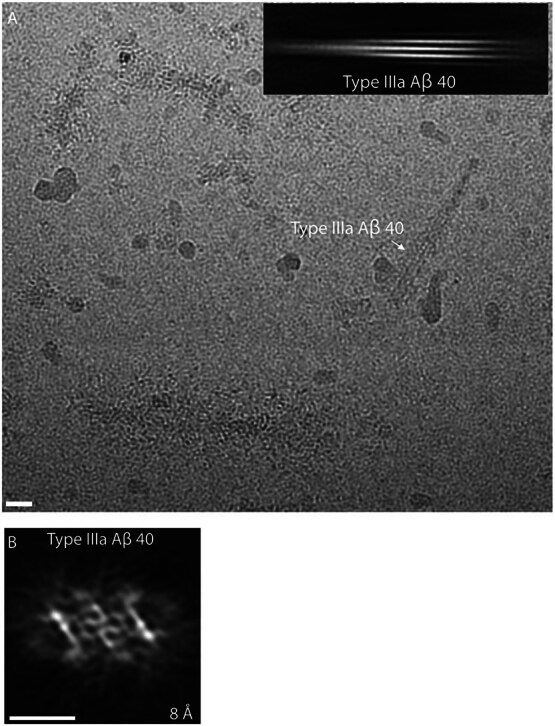
Cryo-EM analysis of Type IIIa Aβ_40_ filaments in DS-1. (**a**) Representative type IIIa Aβ_40_ filaments, indicated in the cryo-EM micrograph with a white arrow, and corresponding 2D class average. (**b**) 3D reconstruction of type IIIa Aβ_40_ filaments. Scale bars represent 10 nm. A total of 12,540 movies were collected for case #1.

Western blot analysis of the sarkosyl-insoluble fractions from the gray matter of DS cases 1 and 2 using antibodies targeted toward Aβ shows the presence of high molecular weight aggregates. Aβ monomers (4 kDa) and dimers (8 kDa) were evident after treatment of the samples with hexafloro isopropanol (HFIP) to disaggregate Aβ fibrils (Extended Data Fig. 2). Mass spectrometric analysis of the same fractions determined the presence of Aβ peptides, predominantly starting at position 1 and 3 and ending at positions 40 and 42 (Extended Data Fig. 2c). We also identified the proteins COL25A1, SMOC1, MDK, NTN1, OLFML3, HTRA1 and EZR in the sarkosyl preparation. These proteins had previously been described to be the most enriched in amyloid plaques of individuals with early-onset AD and DS^32^. Immunogold transmission EM of dispersed preparations of filaments shows Aβ filaments that were decorated by the D54D2 antibody (Extended Data Fig. 2b). We determined the structure of Aβ filaments at high resolution by cryo-EM imaging and three-dimensional (3D) reconstruction. Visual inspection of cryo-EM micrographs shows the presence of Aβ filaments in gray matter of both DS cases (Extended Data Figs. 3 and 4). Two-dimensional (2D) classification of Aβ filaments shows filaments with varying width and crossover distances (Extended Data Figs. 3 and 4). We identified four types of Aβ filaments. Type I and type II Aβ_42_ filaments were present in both DS cases (Fig. 1). Type I Aβ_42_ filaments have a crossover distance of ~30 nm and are composed of two protofilaments packed with a 2_1_ symmetry (Fig. 1). Type I Aβ_42_ filaments, representing ~50–60% of the filaments in both DS cases, were reconstructed to ~3.2 Å resolution. As previously reported for type I Aβ_42_ filaments in AD^29^, the structure extended from Gly_9_ to Ala_42_ in each S-shaped protomer (Fig. 2). Type Ib Aβ_42_ filaments, reported as a small fraction of the total Aβ_42_ filaments in sporadic AD (sAD)^29^, were not observed. Type II Aβ_42_ filaments have a helical twist of ~2.9° and are composed of two protofilaments packed with twofold symmetry. Type II Aβ_42_ filaments, representing ~25–35% of the filaments in both cases, were reconstructed at 4.5 Å resolution (Fig. 1), extending from Val_12_ to Ala_42_ (Fig. 2). Type II Aβ_42_ filaments were identical to the type II Aβ_42_ filaments reported as the predominant type of Aβ_42_ filaments in familial AD (FAD) and other conditions in which Aβ_42_ is present as a copathology, but they seem to be relatively rare in sporadic AD^29^.

### Structure of Aβ filaments ending at Val_40_ (Aβ_40_) in DS

**Fig. 3:**
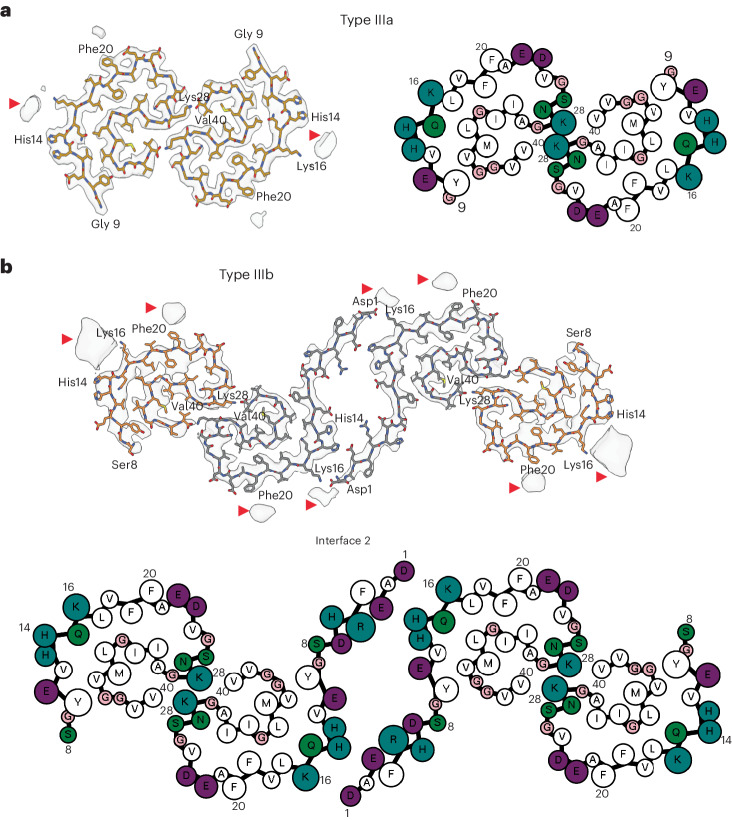
Aβ_40_ filaments in DS brain. **a**,**b**, Atomic model of type IIIa Aβ_40_ (**a**) and type IIIb Aβ_40_ filaments (**b**). Type IIIa Aβ_40_ filaments are made of two identical protofilaments while type IIIb Aβ_40_ filaments are made of two nonidentical protofilaments. Type IIIa Aβ_40_ filaments extend from Gly_9_ to Val_40_, while type IIIb Aβ_40_ filaments extend from Ser_8_ to Val_40_ and from Asp_1_ to Val_40_. Red arrowheads indicate extra densities. Cartoon representation of amino acid residues: hydrophobic (white), positively charged (teal), polar (green), negatively charged (purple) and glycine (pink) residues.

**Fig. 4:**
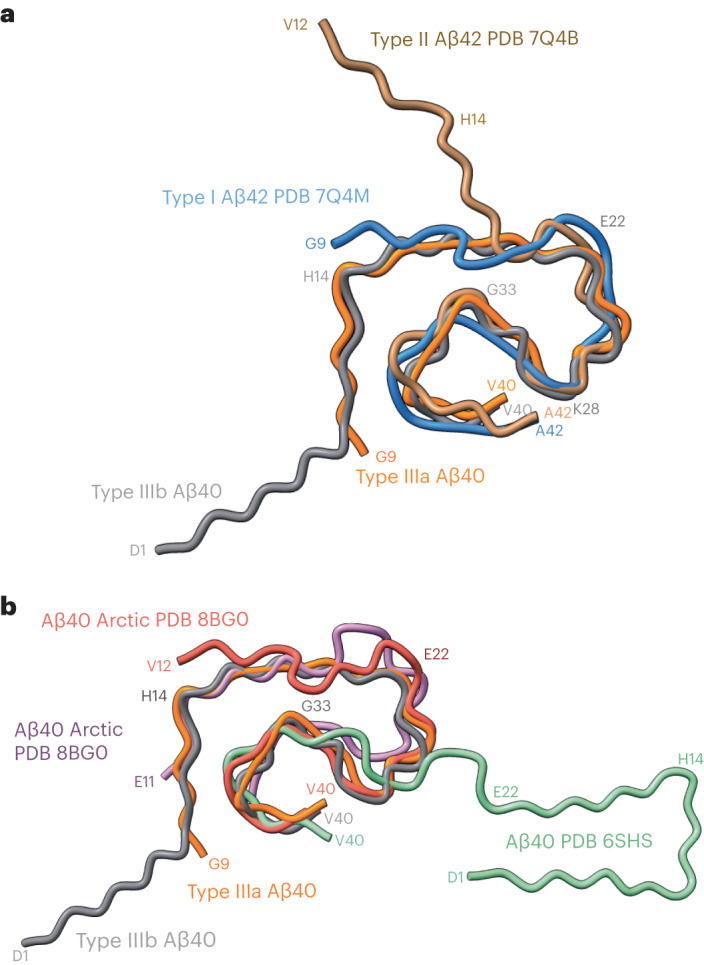
Comparison of type III Aβ_40_ filaments with human Aβ_40_ and Aβ_42_ filaments. **a**, Superposition of type I (blue) and II (brown) Aβ_42_ filaments, and type IIIa (orange) and IIIb (gray) Aβ_40_ filaments based on the central layer of their S-shaped domains. **b**, Superposition of the backbone structures of type IIIa (orange) and IIIb (gray) Aβ_40_ filaments with human Arctic chain A (red) and B (purple) Aβ_40_ filaments, and meningeal (green) human Aβ_40_ filaments.

We reconstructed two additional Aβ filaments in case 2. Type IIIa Aβ_40_ filaments (~3.5 Å resolution) and type IIIb Aβ_40_ filaments (~3.8 Å resolution) represented ~15% of the Aβ filaments (Figs. 1 and 3 and Extended Data Fig. 3). We observed only type IIIa Aβ_40_ filaments, representing ~3% of the filaments, in case 1 (Extended Data Fig. 4). Type IIIa Aβ_40_ filaments have a width of 5–8 nm and are composed of two protofilaments arranged as a dimer with 2_1_ symmetry (interface 1). The core of type IIIa Aβ_40_ filaments is composed of four β-strands that run along the protofilament, adopting a seahorse-shaped figure (Figs. 1, 3 and 4). The two protofilaments of interface 1 pack against each other through van der Waals interactions and a salt bridge between the ε-amino group of Lys_28_ and carbonyl group of Val_40_ (Fig. 3). Type IIIb Aβ_40_ filaments have a width of 12–16 nm and are composed of four protofilaments organized as a dimer of dimers (interface 2), with each dimer similar to the type IIIa Aβ_40_ filaments (Figs. 1 and 3). Residues Gly_9_ to Val_40_ could be modeled into the density maps of type IIIa Aβ filaments and the two outermost protomers of type IIIb Aβ_40_ filaments (Figs. 2 and 3). In the two inner protomers of type IIIb Aβ_40_ filaments, the density at the N-terminal region was resolved due to inter-protofilament interactions, allowing us to model residues Asp_1_ to Val_40_. The outermost protomers of type IIIb Aβ_40_ filaments are composed of five β-strands while the inner protomers are composed of four β-strands (Fig. 2a). At interface 2 of the type IIIb Aβ_40_ filaments, the two dimers interact through their extended N-terminal region through a hydrogen bond between Glu_3_ and His_14_ (Fig. 3). Additional densities around residues His_14_–Lys_16_ and Phe_20_ in both types of III Aβ_40_ filaments were also observed. The extra density around His_14_–Lys_16_ and the density around Phe_20_ may correspond to nonproteinaceous cellular factors. In type IIIb Aβ_40_ filaments, the extra density around His_14_–Lys_16_ coincides with the interaction site between the two innermost protomers of type IIIb Aβ_40_ filaments. This extra density, probably a charged host factor, interacts with both the disordered Asp_1_ and Lys_16_ (Fig. 3). This probable three-way interaction might contribute to the packing of the protofilaments and stabilize the N-terminal region.

**Extended Data Fig. 5:**
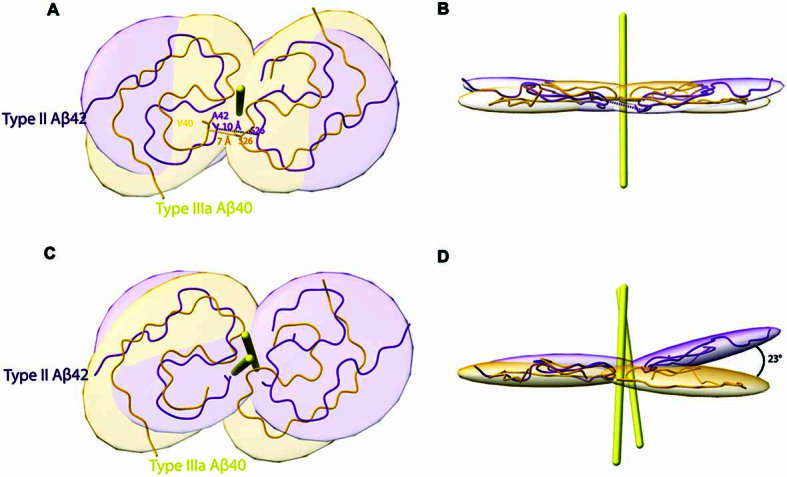
Type III Aβ_40_ is a distinct structure. Superposition of two-fold rotation axes of type IIIa Aβ_40_ (yellow) with type II Aβ_42_ (purple) filaments reveals differences in the packing of residues (**a**, **b**). Superposition of a single chain of type IIIa Aβ_40_ with a single chain of type II Aβ_42_ shows that the two chains are similar, with the second monomer having a residual rotation of 23 ° (**c**, **d**).

At a glance, the overall fold of type III Aβ_40_ filaments closely resembles that of type II Aβ_42_ filaments (Fig. 4a). The inter-protofilament interactions in both type II and type III Aβ filaments is mediated by a salt bridge between Lys_28_ in one protomer and the carbonyl group of the C-terminal residue of the neighboring protomer. However, the C-terminal residues are Ala_42_ in type II Aβ_42_ filaments and Val_40_ in type III Aβ_40_ filaments (Figs. 2 and 3). Closer examination of the structures reveals multiple subtle differences in the packing of residues within the protomers and between the two protofilaments. Superposition of one of the protomers of type III Aβ_40_ filaments with type II Aβ_42_ filaments results in ~23° relative rotation of the other protomer and the inter-protofilament gap in type II filaments is ~2 Å wider than that of the type III Aβ filaments (Extended Data Fig. 5). These differences in rotation of the protomers and inter-protofilament separation allow type II Aβ_42_ filaments to accommodate the two additional C-terminal residues (Ile_41_ and Ala_42_) while maintaining the same salt bridge between Lys_28_ and the terminal carbonyl group in both types of filaments. Considering the presence of different C-terminal residues, nonproteinaceous densities around the filaments, and the distinct helical twist (−2.9° for type II and −0.45° for type III Aβ filaments) (Tables 1 and 2), we conclude that type III Aβ_40_ filaments consist of a unique isoform of Aβ_40_ ending at residue Val_40_. In contrast, type I Aβ_42_ filaments and type II Aβ_42_ filaments are formed by an Aβ_42_ isoform ending at residue Ala_42_ (Figs. 2 and 4a). Comparison of the structure of type III Aβ_40_ filaments with Aβ_40_ filaments having the E22G mutation (Arctic mutation) shows that the presence of the E22G mutation modifies the loop as previously reported in the mutant structure^30^; however, a structural overlap can be noted in the S-shaped C-terminal region (Fig. 4b). Comparison of the structure of type III Aβ_40_ filaments with the structure reported for Aβ_40_ filaments extracted from meninges of individuals with AD^31^ shows some structural similarities on the C-terminal portion of the structure, but a completely different N-terminal structure before ~Ile_31_ (Fig. 4b).

**Table 1:** Cryo-EM data collection, refinement and validation statistics—case 1

	PHF tau	SF tau	Type I Aβ42	Type II Aβ42	Type IIIa Aβ40
**Data collection and processing**					
Magnification	81,000	81,000	81,000	81,000	81,000
Voltage (kV)	300	300	300	300	300
Electron exposure (e^−^ Å^−^^2^)	51.45	51.45	51.45	51.45	51.45
Defocus range (μm)	−0.5 to −1.5	−0.5 to −1.5	−0.5 to −1.5	−0.5 to −1.5	−0.5 to −1.5
Pixel size (Å)	1.054	1.054	1.054	1.054	1.054
Symmetry imposed	C1	C1	C1	C2	C1
Helical rise (Å)	2.38	4.76	2.36	4.78	2.39
Helical twist (°)	179.49	−1.03	178.45	−2.9	179.46
Initial particle images (no.)	1,332,726	1,332,726	233,390	233,390	233,390
Final particle images (no.)	208,054	101,890	34,396	22,740	6,156
Map resolution (Å)	3.3	3.2	3.3	5.0	8.1
FSC threshold	0.143	0.143	0.143	0.143	0.143

**Table 2:** Cryo-EM data collection, refinement and validation statistics—case 2

	PHF tau (EMDB 40411), (PDB 8SEH)	SF tau (EMDB 40413), (PDB 8SEI)	Type I Aβ42 (EMDB 40416), (PDB 8SEJ)	Type IIIa Aβ40 (EMDB 40419), (PDB 8SEK)	Type IIIb Aβ40 (EMDB 40421), (PDB 8SEL)
**Data collection and processing**					
Magnification	81,000	81,000	81,000	81,000	81,000
Voltage (kV)	300	300	300	300	300
Electron exposure (e^−^ Å^−^^2^)	52.35	52.35	52.35	52.35	52.35
Defocus range (μm)	−0.5 to −1.5	−0.5 to −1.5	−0.5 to −1.5	−0.5 to −1.5	−0.5 to −1.5
Pixel size (Å)	1.054	1.054	1.054	1.054	1.054
Symmetry imposed	C1	C1	C1	C1	C2
Helical rise (Å)	2.38	4.78	2.38	2.39	4.76
Helical twist (°)	179.46	−1.04	178.24	179.77	−0.46
Initial particle images (no.)	2,636,429	2,636,429	17,239,321	17,239,321	17,239,321
Final particle images (no.)	90,384	21,743	45,575	5,982	4,295
Map resolution (Å)	2.9	2.9	3.17	3.5	3.8
FSC threshold	0.143	0.143	0.143	0.143	0.143
**Refinement**					
Initial model used (PDB code)	5O3L	5O3T	7Q4B	7Q4M	7Q4M
Model resolution (Å)	3.2	2.9	3.5	3.7	4.3
FSC threshold	0.5	0.5	0.5	0.5	0.5
Map sharpening *B* factor (Å^2^)	−63	−70	−110	−120	−110
Model composition					
Nonhydrogen atoms	5,570	5,570	2,510	2,380	5,510
Protein residues	730	730	340	320	730
Root mean square deviations					
Bond lengths (Å)	0.026	0.026	0.034	0.032	0.026
Bond angles (°)	1.902	1.828	1.695	2.250	2.100
**Validation**					
MolProbity score	0.95	1.32	1.06	1.60	1.44
Clashscore	0	1.23	1.39	1.34	1.75
Poor rotamers (%)	0	0	0	0	0
Ramachandran plot					
Favored (%)	92.96	92.25	96.88	80.00	92.75
Allowed (%)	7.04	7.04	3.12	20.00	4.35
Disallowed (%)	0	0	0	0	2.90

### Structures of tau filaments in DS

**Fig. 5:**
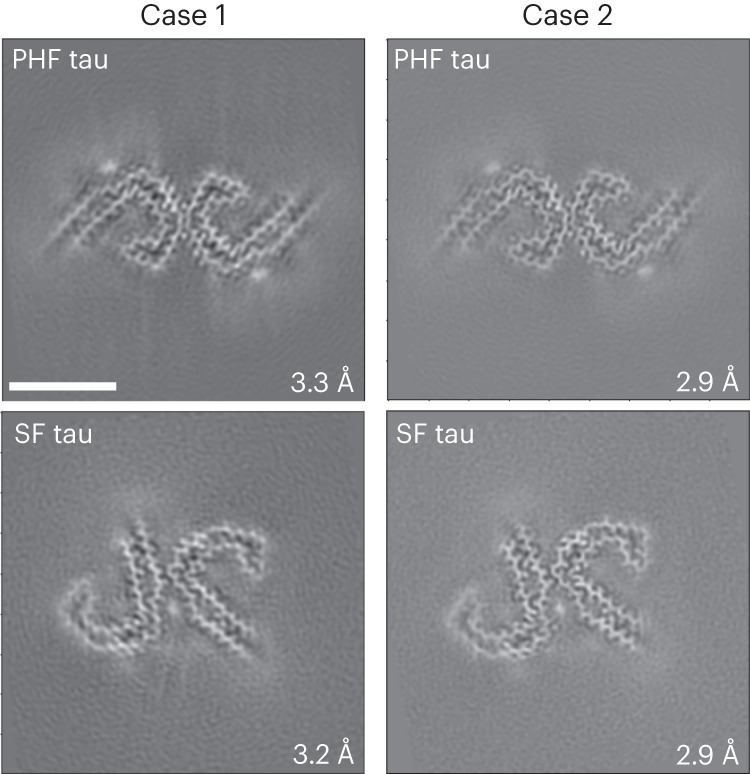
Cryo-EM reconstructions of tau filaments. Cryo-EM maps, depicted as central slices, of tau filaments from the two DS cases (1 and 2). The structures show identical pairs of C-shaped protofilaments and the symmetric inter-protofilament packing between PHFs and asymmetric packing for SFs. The estimated resolution is shown on the bottom right. Scale bar, 10 nm. A total of 12,540 and 24,881 movies were collected for cases 1 and 2, respectively.

**Extended Data Fig. 6:**
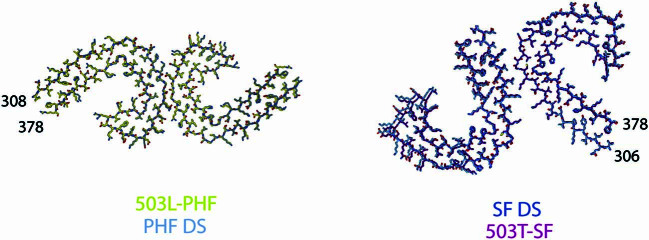
Atomic model of tau in DS. Map fit in the model for PHF and SF from DS compared to PHFs and SF in AD (33).

Western blot analysis of the sarkosyl-insoluble fractions from the gray matter of cases 1 and 2 using the anti-tau antibody HT7 shows the presence of tau bands with a migration pattern corresponding to 3+4R tau, with identical electrophoretic mobility in both cases (Extended Data Fig. 2). Mass spectrometric analysis of the same fractions determined the presence of tau tryptic peptides, with 100% coverage of the filament’s core region. Tau filaments are highly post-translationally modified, especially in respect to phosphorylation, acetylation, ubiquitination and deamidation. A number of proteins were identified in both DS individuals that co-purified with the tau preparation, including 24 unique proteins previously identified as part of the tau interactome in AD^33^. Immunogold transmission EM of dispersed preparations of tau filaments shows phosphorylated twisted ribbon filaments that were decorated by the AT8 antibody (Extended Data Fig. 2). We determined the structure of tau filaments at high resolution by cryo-EM imaging and 3D reconstruction (Fig. 5). Visual inspection of cryo-EM micrographs shows the presence of paired helical filaments (PHFs) and straight filaments (SFs) in both cases (Extended Data Fig. 3), identical to those of PHFs and SFs from AD and related diseases (Extended Data Fig. 6)^34–36^. We determined the structure of PHFs to a resolution of 2.9 Å and the structure of SFs to a resolution of 2.9 Å (Fig. 5). PHFs and SFs were similarly present in the DS preparations.

## Discussion

Type I Aβ_42_ filaments have been previously reported as the predominant type of filaments in cases of sAD with abundant parenchymal Aβ deposition in the form of core plaques, while type II Aβ_42_ filaments were found predominantly in cases of FAD (*AβPP V717F* and *PSEN1 F105L* mutations), as well as in other neurodegenerative diseases, in association with parenchymal Aβ_42_ deposition in the form of more diffuse deposits^29^. In the two DS cases, we observed both types of Aβ_42_ filaments, present at similar levels. In DS, Aβ plaque density and morphology may be like that seen in sAD^5^; however, subtle regional differences in amyloid deposition have been reported between AD in DS, sAD and FAD^37^. Aβ deposition occurs many decades earlier in DS compared to sAD, with diffuse deposits being reported in people with DS between 8 and 27 years of age^9–11,19,20^. We studied two DS individuals aged 46 and 59 years. At this age, it is considered that the level of cortical Aβ deposition is similar to that observed in sAD^5^. In both cases, we observed the presence of senile plaques and abundant diffuse deposits, containing Aβ_42_ peptides that were recognized using antibodies against peptides ending at position Ala_42_ (Extended Data Fig. 1). Our cryo-EM study shows the presence of type I and II Aβ_42_ filaments, probably reflecting the presence of parenchymal core plaques and diffuse deposits in the two DS individuals. Both DS cases were carriers of one *APOE ε4* allele, with previous work suggesting that the presence of one *APOE ε4* allele makes no difference on the structure of Aβ_42_ filaments in sAD^29^. The *APOE ε4* allele is the most established genetic risk factor for sAD^38^ and may also play a role in the risk and age at onset of dementia in DS^39^. Interestingly, on a large cohort of adults with DS with clinical assessments and multimodal biomarkers, it has recently been shown that the *APOE ɛ4* allele exerts a similar association with AD pathophysiological processes in DS as in the general population^40^. These studies emphasize the similarities between the mechanisms at play in amyloid aggregation in AD and AD in DS.

**Extended Data Fig. 7:**
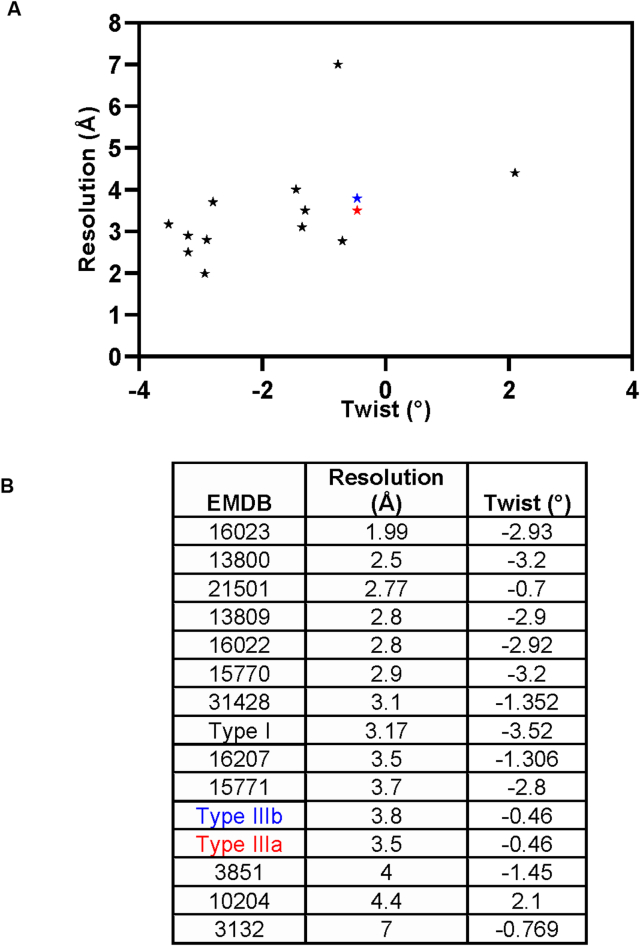
Twist values of Aβ filaments shown in a scatter plot (A) and table (B). Data points for the DS type IIIa and IIIb Aβ_40_ filaments are colored in red and blue, respectively.

While parenchymal Aβ deposition involves mostly Aβ peptides ending at Ala_42_, vascular Aβ deposition involves mostly Aβ peptides ending at Val_40_. CAA has been observed to be present at significantly higher frequencies and severity in the brains of individuals with DS compared to sAD cases^19,20^. Both DS cases showed CAA containing Aβ_40_ peptides that were recognized using antibodies against peptides ending at position Val_40_ (Extended Data Fig. 1), and the presence of Aβ_40_ filaments. Cryo-EM studies have recently revealed the structure of Aβ amyloid fibrils ending at position 40 from the vasculature (meninges) of AD brain reconstructed at 4.4 Å resolution, with a right-handed twist (Extended Data Fig. 7; EMDB ID: 10204)^31^. We were unable to observe the three fibril structures reported by Kollmer et al.^31^ but rather determined two structures composed of Aβ_40_ filaments formed by Aβ peptides ending at position Val_40_, types IIIa and IIIb Aβ_40_ filaments, which were reconstructed at 3.5 and 3.8 Å resolution, respectively (Fig. 1). Interestingly, we observed a structural overlap, which can be noted in the S-shaped C-terminal region, between types IIIa and IIIb Aβ_40_ filaments and Aβ_40_ filaments having the Arctic mutation at position 22 (E22G) of Aβ_40_ (ref. ^30^). The relevance of these structural differences between Aβ_40_ filaments is unknown, and further research is needed to determine whether type IIIa and IIIb Aβ_40_ filaments are unique to DS or are also present in sporadic CAA and in CAA associated with AD.

In addition to parenchymal and vascular amyloid pathology, individuals with DS develop tau pathology, with a similar pattern to tau pathology in AD^5–15^. We showed that there is no variation in the structure of tau filaments between individuals with DS and sAD (and FAD), and tau in other brain amyloidosis^34–36^, further supporting the notion of a common mechanism through which different amyloids trigger aggregation of tau, resulting in tau filaments with identical structure at their core^41^. Our data suggest that DS, which may be considered a genetic form of AD, shares common pathogenic mechanisms with sAD and FAD, leading to parenchymal and vascular amyloid deposition and tau aggregation. Knowledge of similarities and differences between sAD, FAD and AD in DS are crucial for understanding these disorders and assessing whether adults with DS could be an optimal population in whom to conduct AD prevention trials.

## Methods

### Clinical history and neuropathology

Human tissue samples were from the Dementia Laboratory Brain Library at Indiana University School of Medicine. Their use in this study was approved by the ethical review processes at the institution. Informed consent was obtained from patients’ next of kin.

We studied two individuals with Down syndrome. Case 1 (DS-1) was a 59-year-old male who died with neuropathologically confirmed diagnosis of AD after a 6 year history of progressive dementia. He also had a clinical history of a seizure disorder with multifocal myoclonic jerks. A brain autopsy was carried out. The fresh brain weighed 750 g with severe atrophy in the frontal and temporal lobes. Moderate to severe neuronal loss and gliosis were seen in the frontal cortex, temporal cortex, parietal cortex, hippocampus, thalamus, midbrain, pons, medulla and cerebellum. Numerous plaques and neurofibrillary tangles were present in these same areas. The AD neuropathologic change was ranked as high AD neuropathologic change with Thal phase 5 (A3), Braak stage VI (B3) and Consortium to Establish a Registry for Alzheimer’s Disease score of C3 (A3, B3 and C3). Moderate CAA was present. Case 2 (DS-2) was a 46-year-old male who died with neuropathologically confirmed diagnosis of AD after over a 6 year history of progressive dementia. Both his mother and maternal grandmother had a history of dementia. A brain autopsy was carried out. The fresh brain weighed 1,056 g. There was mild symmetric atrophy, with mild to moderate atrophy of the left cerebral hemisphere. The left and right hemibrains weighed 526 and 516 g, respectively. Mild atheromatous change was present in the middle cerebral arteries. Mild to moderate numbers of neuritic plaques were observed in the neocortex, amygdala, hippocampus, entorhinal cortex and midbrain. NFTs, neuronal loss, gliosis and moderate to severe CAA were present. The AD neuropathologic change was ranked as intermediate AD neuropathologic change with Thal phase 3 (A2), Braak stage IV (B2) and Consortium to Establish a Registry for Alzheimer’s Disease score of C2 (A3, B2 and C2). Tissue samples for neuropathological studies were obtained from representative brain regions. The 8 μm thick brain sections were used. For immunohistochemistry, primary antibodies were 4G8 (Abcam, 1:1,000), β-amyloid 1–40 (Millipore Sigma, 1:400), β-amyloid 1–42 (Millipore Sigma, 1:400) and AT8 (anti-phospho tau, Thermo Fisher, 1:300). The signal from the antibodies was visualized using avidin–biotin followed by horseradish peroxidase-conjugated streptavidin and the chromogen diaminobenzidine. Immunohistochemical sections were counterstained with hematoxylin.

### Genetics

To confirm trisomy 21, we performed chromosomal microarray analysis on genomic DNA extracted from brain using a whole genome platform that includes both nonpolymorphic and single-nucleotide polymorphism oligonucleotide probes (Affymetrix CytoScan HD Microarray). Patient hybridization results were compared to data pooled from hundreds of normal individuals. To assess for common copy number variations (CNVs) in the populations and regions of clinical significance, databases potentially consulted include, but are not limited to, the International Standards for Cytogenomic Arrays Clinical CNV Database, Database of Genomic Variants, DECIPHER Population CNV Database, Online Mendelian Inheritance in Man, ClinGen, ClinVar and PubMed. All results are analyzed and reported using the February 2009 National Center for Biotechnology Information human genome build 37.1 (hg19).

### Filament extraction

Sarkosyl-insoluble fractions were prepared from freshly frozen frontal cortex of DS-1 and temporal cortex of DS-2, as previously described^36,42^. Briefly, ~2 g of tissue was homogenized in 20 volumes (w/v) extraction buffer consisting of 10 mM Tris–HCl, pH 7.4, 0.8 M NaCl, 1 mM EGTA and 10% sucrose. Samples were centrifuged at 20,000*g* and the supernatants were brought to 2% sarkosyl and incubated at 37 °C for 1 h. Samples were centrifuged at 10,000*g* for 10 min. The supernatants were spun at 100,000*g* for 1 h at 4 °C. The resulting pellets were resuspended in 1 ml g^−1^ tissue in the extraction buffer and centrifugated at 3,000*g* for 5 min. This supernatant was further purified by threefold dilution in buffer consisting of 50 mM Tris–HCl, pH 7.5, 0.15 M NaCl, 10% sucrose and 0.2% sarkosyl, followed by centrifugation at 100,000*g* for 30 min at 4 °C. The final pellet was resuspended in 20 mM Tris–HCl, pH 7.5 and 50 mM NaCl and stored at 4 °C.

### Western blotting

Samples were sonicated for 1 min, boiled with gel 2× Laemmli sample buffer (Bio-Rad) for 5 min at 100 °C and resolved on 4–12% Bis-Tris gels for Aβ-amyloid or 10% Bis-Tris gels for tau (NuPAGE). For HFIP treatment, samples were centrifuged at 200,000*g* for 30 min and HFIP added to the pellet, sonicated and left overnight at 37 °C. Samples were dried under nitrogen, washed three times with water and centrifuged at 200,000*g* for 30 min. The final pellet was resuspended in loading buffer and resolved on 4–12% Bis-Tris gels. Proteins were transferred to nitrocellulose membranes and the membranes were incubated with blocking solution (5% nonfat milk in phosphate-buffered saline with 0.1% Tween 20). Membranes were incubated for 1 h with primary antibody diluted in TBS. Antibodies used were 4G8 (BioLegend, 1:1,000), 6E10 (BioLegend, 1:1,000), AT8 (Thermo Fisher, 1:1,000) and HT7 (Thermo Fisher, 1:1,000). After incubation with secondary antibody for 45 min, proteins were visualized using a chemiluminescence kit (SuperSignal West Pico, ThermoFisher) according to the manufacturer’s specifications.

### Immuno-EM

For immuno-EM, samples were analyzed as previously described^28,36^. AT8 antibody or D5452 antibody (anti-amyloid-β, Cell Signaling) were diluted 1:50 in 0.1% gelatin in phosphate-buffered saline and incubated overnight at 4 °C. Secondary antibodies used were 6 nm anti-mouse and 10 nm anti-rabbit immunogold particles (Electron Microscopy Sciences). Negative staining was performed with NanoVan (Ted Pella) for 5 s at room temperature. Images were taken on a Tecnai G2 Spirit Twin scope equipped with an AMT CCD Camera.

### Mass spectrometry sample preparation

Samples were diluted in 8 M urea, 50 mM Tris–HCl pH 8.5 (100 µl), reduced with 5 mM Tris(2-carboxyethyl)phosphine hydrochloride for 30 min at 37 °C and alkylated with 10 mM chloroacetamide at room temperature in the dark, for 30 min. Samples were digested in two steps with LysC/trypsin (Promega). After overnight trypsin digestion in 2 M urea, the samples were applied to Pierce detergent removal spin columns (Thermo Scientific) and then desalted on SepPak 18 cartridge (Waters Corporation) washed with 1 ml of 0.1% trifluoroacetic acid, eluted in 600 µl of 70% acetonitrile/0.1% formic acid (FA) and dried by speed vac.

### Liquid chromatography with tandem mass spectrometry

Samples were reconstituted in 50 µl of 0.1% FA and 7 µl were injected on an Easynano LC1200 coupled with Aurora 25 cm column (IonOpticks) insonation column oven (40 °C) on an Eclipse Orbitrap mass spectrometer (Thermo Fisher Scientific). Peptides were eluted on a 115 min gradient from 5% to 35% B, increasing to 95% B over 10 min and decreasing to 5% B for 5 min (solvent A: 0.1% FA; solvent B: 80% acetonitrile, 0.1% FA). The instrument was operated with FAIMS pro 4 coefficients of variation (−30, −45, −55 and −65 V), positive mode, 0.6 s cycle time per coefficient of variation with APD and Easy-IC on. Full scan included 400–1,500 *m*/*z* with 60,000 resolution, standard automatic gain control and auto max IT, 40% RF lens, 5 × 10^4^ intensity threshold, charge states 2–8 and 30 s dynamic exclusion with common settings. MS2 parameters of 1.6 *m*/*z* quadrupole isolation, 30% fixed higher-energy collision dissociation cell, 15,000 Orbitrap resolution, standard automatic gain control and dynamic IT were included.

### Mass spectrometry data analysis

Raw files were loaded into PEAKS X Pro Studio 10.6 Build 20201221 (Bioinformatics Solutions). The precursor ion tolerance was 10 ppm 0.02 Da. Peptides obtained after trypsin digestion were used for database searches of the reviewed Uniprot_Swissprot *Homo sapiens* database and common contaminants (20,437 entries) with variable post-translational modifications (PTMs). PEAKS PTM and SPIDER searches were enabled to search all de novo peptides above a 15% score for over 300 potential PTMs and mutations. A 0.1% peptide false discovery rate cutoff (−10 log *P* ≥21.8), PTM A score >10, mutation ion intensity >1 and de novo only score >80% were applied to the data, followed by PEAKS LFQ analysis. Raw and searched data are available at ProteomeXchange. The bioinformatic analysis Gene Ontology of identified proteins was done by DAVID Bioinformatics Resources 6.8 (refs. ^43,44^). *P* value was represented as −log_10_. The Venn diagram was generated using BioVenn^45^.

### High‑resolution cryo‑EM imaging

Cryo-EM grids of brain extracts of the two patients with DS were prepared in a biosafety level 2 cabinet while wearing appropriate personal protective equipment. A total of 2–3 µl of the sample were applied on a graphene oxide-coated EM grid, then washed with 10 mM Tris pH 7.8 before vitrifying using a semi-automated Gatan CP3 cryo-plunger. High-resolution cryo-EM movies were collected on a FEI Titan Krios at 300 kV with a Gatan K3 detector mounted on a quantum energy filter with 20 eV slit width (Tables 1 and 2). For DS-1, we recorded 12,540 movies of 50 frames per movie with an exposure time of 48 ms per frame, with a dose rate of 21 electrons per Å^2^ per frame for a total accumulated dose of 51.45 electrons per Å^2^ at a pixel size of 1.054 Å. For DS-2, we recorded 24,881 movies of 50 frames per movie with an exposure time of 52 ms per frame and a dose rate of 1.054 electrons per Å^2^ per frame for a total accumulated dose of 52.35 electrons per Å^2^ at a pixel size of 1.054 Å. The datasets were collected with defocus values ranging from −0.5 to −1.5 μm. The movies were gain corrected, motion corrected and dose weighted using MotionCor2 (ref. ^46^). The contrast transfer function of all aligned and non-dose-weighted micrographs was estimated using CTFFIND-4.1 (ref. ^47^).

### Helical reconstruction

**Extended Data Fig. 8:**
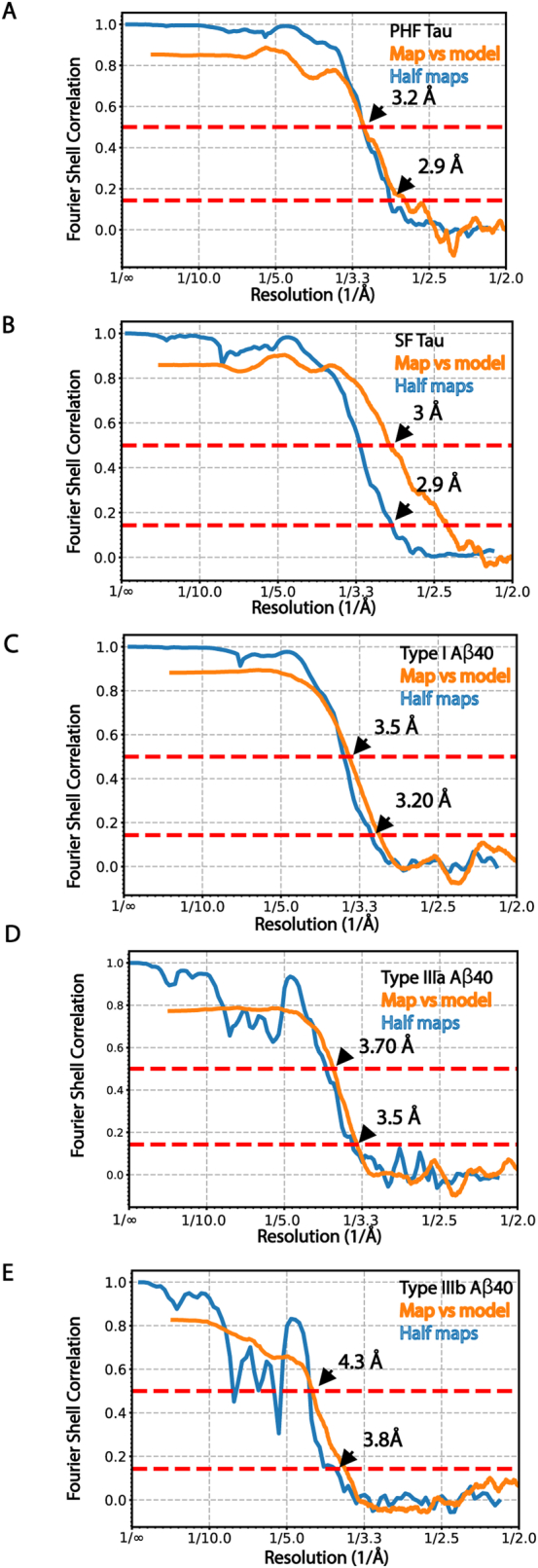
Fourier shell correlation curves. Fourier shell correlation (FSC) curves for independently refined two half maps and map vs model for PHF tau (**a**), SF tau (**b**), type I (**c)**, IIIa (**d**) and IIIb (**e**) Aβ filaments. The FSC threshold of 0.143 and 0.5 are shown as blue and orange line respectively.

Image processing was performed in RELION 4.0 (ref. ^48^). Filaments were picked manually for DS-1 and automatically for DS-2 using RELION helical picker as end-to-end line segments. The initial quantity of particles for DS-2 was excessively high from the automatic over picking to ensure that all filaments were picked. We extracted all the helical segments with a box size of 600 pixels (632 Å), downscaled to 200 pixels to speed up analysis, and an inter-box distance of ~15 Å. Several rounds of reference-free 2D classifications were carried out to remove nonfilament contaminants and to find homogeneous subsets using a regularization value of *T* = 1–2. Aβ and tau filaments were visually identified from the 2D class averages. For both, a new 256 pixel box size, without downscaling, was used to re-extract the filament segments with inter-box distance of approximately 15 Å. The initial 3D reference maps were reconstructed de novo from best 2D class averages comprising a full helical crossover using relion_helix_inimodel2d. The initial round of 3D classification was low-pass filtered to 10 Å. Several rounds of 3D classification were carried out to obtain the best homogeneous subset. The final selected segments were used for final 3D auto-refinement with optimization of the helical twist and rise to yield a 3D map showing clearly visible β-strand separation and side-chain densities. For the less abundant type III filaments, we first reconstructed the type IIIb filaments consisting of a dimer of dimer packing of the protofilaments. For type IIIa filaments, which have a pitch similar to that of type IIIb filaments but are narrower, we assumed that the type IIIa filaments corresponded to one of the two dimers in the type IIIb filaments. A dimer (for example, half of the type IIIb filament) was segmented, centered and examined using HI3D^49^, which showed that the two protofilaments in the dimers are packed with a 2_1_ screw symmetry instead of C2 symmetry. These analyses helped obtain the initial model and helical parameters to reconstruct the type IIIa filament structure. Bayesian polishing was subsequently applied, followed by contrast transfer function refinement. A 3D classification was done to remove suboptimal segments, along with another round of 3D auto-refinement with optimization of the helical twist and rise. We used a 10–30% *z* percentage to generate the mask for post-processing and resolution estimation. The final reconstructions were sharpened using the standard post-processing procedures in RELION. The overall resolution was calculated from Fourier shell correlations at 0.143 between two independently refined half-maps, employing phase randomization for the convolution effects correction of an optimized, soft-edged solvent mask as implemented in the trueFSC.py program in JSPR software (Extended Data Fig. 8)^50^.

### Atomic modeling and structural analysis

**Extended Data Fig. 9:**
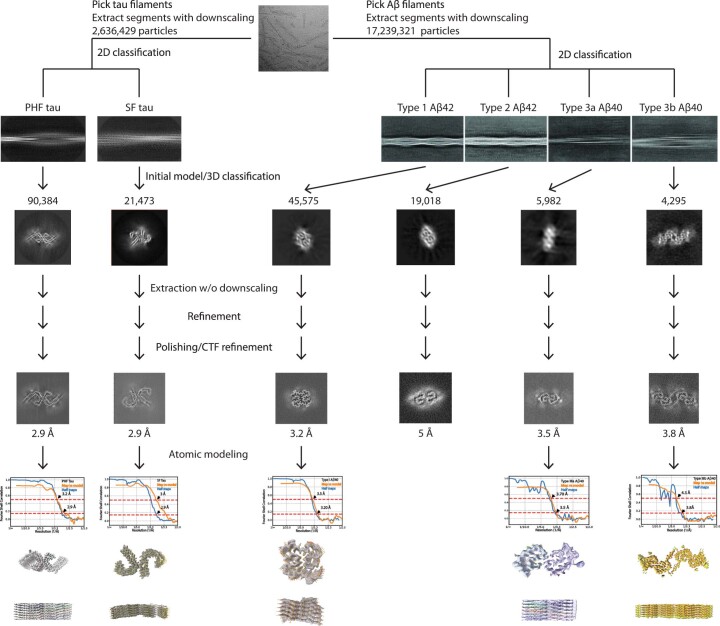
Cryo-EM image analysis of Down syndrome amyloids. The diagram shows the workflow of image analysis and 3D reconstruction of the two types of tau filaments, and four types of Aβ filaments using RELION 4.0.

The previously deposited model was fitted into the sharpened density maps using ChimeraX^51^. The central chain of each model was manually adjusted in Coot^52^. The atomic positions of all models were refined with their respective helical symmetry parameters using Rosetta^53^. The identity and the sequence range of the modeled proteins were validated using the map2seq Web app^54^. Final atomic models were validated using MolProbity^55^ (Tables 1 and 2). All models’ figures were generated in ChimeraX^51^ (Extended Data Fig. 9).

### Reporting summary

Further information on research design is available in the Nature Portfolio Reporting Summary linked to this article.

## Online content

Any methods, additional references, Nature Portfolio reporting summaries, source data, extended data, supplementary information, acknowledgements, peer review information; details of author contributions and competing interests; and statements of data and code availability are available at 10.1038/s41594-024-01252-3.

### Supplementary information

Reporting Summary

### Source data

Source Data Extended Data Fig. 2 Raw data blots for Extended Data Fig. 2.

## Data Availability

Cryo-EM maps have been deposited in the Electron Microscopy Data Bank (EMDB) under accession numbers EMD-40411, 40413, 40416, 40419 and 40421. Refined atomic models have been deposited in the Protein Data Bank (PDB) under accession numbers PDB 8SEH (tau), 8SEI (tau), 8SEJ (type I Aβ), 8SEK (type IIIa Aβ) and 8SEL (type IIIb Aβ). The mass spectrometry proteomics data generated in this study have been deposited to the MassIVE repository with the dataset identifier MSV000091451. Source data are provided with this paper.
