# Supplementary material for: Cryo-EM structures of amyloid-β and tau filaments in Down syndrome
Source: Nat Struct Mol Biol. 2024 Mar 29;31(6):903–9. doi: 10.1038/s41594-024-01252-3 (PMC11189299; doi:10.1038/s41594-024-01252-3)
Supplement: Supplementary file 1 [file 41594_2024_1252_MOESM1_ESM.pdf]

nature portfolio

Corresponding author(s): Ruben Vidal

Last updated by author(s): 11/22/2023

Reporting Summary

Nature Portfolio wishes to improve the reproducibility of the work that we publish. This form provides structure for consistency and transparency in reporting. For further information on Nature Portfolio policies, see our Editorial Policies and the Editorial Policy Checklist.

Statistics

For all statistical analyses, confirm that the following items are present in the figure legend, table legend, main text, or Methods section.

n/a	Confirmed	
☐	☒	The exact sample size (*n*) for each experimental group/condition, given as a discrete number and unit of measurement
☐	☒	A statement on whether measurements were taken from distinct samples or whether the same sample was measured repeatedly
☒	☐	The statistical test(s) used AND whether they are one- or two-sided<br>*Only common tests should be described solely by name; describe more complex techniques in the Methods section.*
☒	☐	A description of all covariates tested
☒	☐	A description of any assumptions or corrections, such as tests of normality and adjustment for multiple comparisons
☒	☐	A full description of the statistical parameters including central tendency (e.g. means) or other basic estimates (e.g. regression coefficient) AND variation (e.g. standard deviation) or associated estimates of uncertainty (e.g. confidence intervals)
☒	☐	For null hypothesis testing, the test statistic (e.g. *F*, *t*, *r*) with confidence intervals, effect sizes, degrees of freedom and *P* value noted<br>*Give P values as exact values whenever suitable.*
☒	☐	For Bayesian analysis, information on the choice of priors and Markov chain Monte Carlo settings
☒	☐	For hierarchical and complex designs, identification of the appropriate level for tests and full reporting of outcomes
☒	☐	Estimates of effect sizes (e.g. Cohen's *d*, Pearson's *r*), indicating how they were calculated

Our web collection on statistics for biologists contains articles on many of the points above.

Software and code

Policy information about availability of computer code

Data collection	CryoEM data were collected in a Ttian Krios G4 using EPU v2.14
Data analysis	Data were analyzed using MotionCor2 v2.1, CTFFIND-4.1, RELION v4.0, trueFSC.py in JSPR software v2023-02-08, Chimera v1.17.1 ChimeraX v1.7, Coot v1.0.05, Rosetta v3.13, map2seq at https://jiang.bio.purdue.edu/map2seq, ProCart at https://jiang.bio.purdue.edu/procart, HI3D at https://jiang.bio.purdue.edu/hi3d.

For manuscripts utilizing custom algorithms or software that are central to the research but not yet described in published literature, software must be made available to editors and reviewers. We strongly encourage code deposition in a community repository (e.g. GitHub). See the Nature Portfolio guidelines for submitting code & software for further information.

Data

Policy information about availability of data

All manuscripts must include a data availability statement. This statement should provide the following information, where applicable:

- Accession codes, unique identifiers, or web links for publicly available datasets
- A description of any restrictions on data availability
- For clinical datasets or third party data, please ensure that the statement adheres to our policy

Cryo-EM maps have been deposited in the Electron Microscopy Data Bank (EMDB) under accession numbers EMD-40411, 40413, 40416, 40419, 40421. Refined

atomic models have been deposited in the Protein Data Bank (PDB) under accession numbers PDB-8SEH, 8SEI, 8SEJ, 8SEK, 8SEL. The mass spectrometry proteomics data generated in this study have been deposited to the MassIVE repository with the dataset identifier MSV000091451.

Research involving human participants, their data, or biological material

Policy information about studies with human participants or human data. See also policy information about sex, gender (identity/presentation), and sexual orientation and race, ethnicity and racism.

Reporting on sex and gender	N/A
Reporting on race, ethnicity, or other socially relevant groupings	N/A
Population characteristics	See Methods section. Case #1 (DS-1) was a 59-year-old male. Case #2 (DS-2) was a 46-year-old male. Both had neuropathologic diagnosis of AD. Trisomy 21 was verified by chromosomal microarray analysis on genomic DNA from brain tissue.
Recruitment	Samples were selected based on neuropathological examination and tissue availability. The selection did not impact the results.
Ethics oversight	The studies carried out at Indiana University were approved through the institutional review process at the University. Informed consent was obtained from the patients' next of kin. Participants did not receive compensation.

Note that full information on the approval of the study protocol must also be provided in the manuscript.

Field-specific reporting

Please select the one below that is the best fit for your research. If you are not sure, read the appropriate sections before making your selection.

☒ Life sciences ☐ Behavioural & social sciences ☐ Ecological, evolutionary & environmental sciences

For a reference copy of the document with all sections, see nature.com/documents/nr-reporting-summary-flat.pdf

Life sciences study design

All studies must disclose on these points even when the disclosure is negative.

Sample size	Frontal cortex from case #1 (DS-1) and temporal cortex from case #2 (DS-2). Samples were chosen based on availability of tissue (maximun available sample size). Cryo-EM data collections continued until reaching the number of amyloid fibrils deemed sufficient to reconstruct amyloid fibrils at ~3 Å resolution.
Data exclusions	Particle images were selected with the highest resolution content in the cryo-EM reconstruction process. Details are presented in Table 1. All movies without any filaments were excluded from the analysis. In addition, particles in poor-quality 2D class averages and 3D classes were discarded.
Replication	At least 3 independent biological repeats per experiment where representative data is show. All replication experiments were successful.
Randomization	N/A. Samples (brain areas) were selected based on neuropathological examination.
Blinding	N/A.

Reporting for specific materials, systems and methods

We require information from authors about some types of materials, experimental systems and methods used in many studies. Here, indicate whether each material, system or method listed is relevant to your study. If you are not sure if a list item applies to your research, read the appropriate section before selecting a response.

Materials & experimental systems

n/a	Involved in the study
☐	☒ Antibodies
☒	☐ Eukaryotic cell lines
☒	☐ Palaeontology and archaeology
☒	☐ Animals and other organisms
☒	☐ Clinical data
☒	☐ Dual use research of concern
☒	☐ Plants

Methods

n/a	Involved in the study
☒	☐ ChIP-seq
☒	☐ Flow cytometry
☒	☐ MRI-based neuroimaging

Antibodies

Antibodies used

Primary antibodies were 4G8 (Biolegend, 1:1000), 6E10 (Biolegend, 1:1000), AT8 (Thermo Fisher, 1:1000), HT7 (Thermo Fisher, 1:1000), and D5452 antibody (anti-amyloid beta, Cell Signaling).

Validation

All commercial (Biolegend, ThermoFisher, Cell Signaling). 4G8 and 6E10 (Biolegend) were validated by wester blot, IHC and ELISA analyses. AT8 and HT7 (Thermo Fisher) were validated by wester blot, IHC and ELISA analyses. D54D2 was validated by western blot analysis of human Aβ-42, Aβ-40, Aβ-39, Aβ-38, and Aβ-37 peptides.
