# Supplementary material for: Cryo-EM structures of amyloid-β and tau filaments in Down syndrome
Source: Nat Struct Mol Biol. 2024 Mar 29;31(6):903–9. doi: 10.1038/s41594-024-01252-3 (PMC11189299; doi:10.1038/s41594-024-01252-3)
Supplement: Supplementary file 2 [file 41594_2024_1252_MOESM2_ESM.pdf]

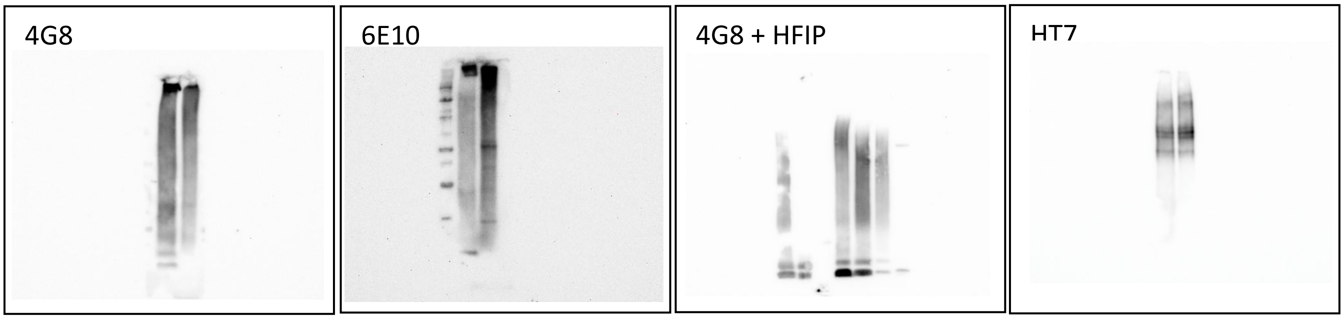
4G8
6E10
4G8 + HFIP
HT7
